# Dihydrotestosterone (DHT) Enhances Wound Healing of Major Burn Injury by Accelerating Resolution of Inflammation in Mice

**DOI:** 10.3390/ijms21176231

**Published:** 2020-08-28

**Authors:** Huaikai Shi, Tsun-Ho Lo, Duncan Ma, Brenton Condor, Brian Lesmana, Roxanne J Parungao, Kevin H.-Y. Tsai, Sarah Kim, Hsiao-Ting Chen, Pablo A Silveira, Zhe Li, Mark S Cooper, Ulla Simanainen, David J Handelsman, Peter K Maitz, Yiwei Wang

**Affiliations:** 1 Burns Research and Reconstructive Surgery, ANZAC Research Institute, University of Sydney, Sydney 2139, Australia; hshi8601@uni.sydney.edu.au (H.S.); duncanwxma@gmail.com (D.M.); brenton.condor@student.unimelb.edu.au (B.C.); bles5603@uni.sydney.edu.au (B.L.); rpar4161@uni.sydney.edu.au (R.J.P.); zhe.li1@sydney.edu.au (Z.L.); peter.maitz@sydney.edu.au (P.K.M.); 2 Dendritic Cell Research, ANZAC Research Institute, University of Sydney, Sydney 2139, Australia; kevin.lo@svha.org.au (T.-H.L.); hche4003@uni.sydney.edu.au (H.-T.C.); pablo.silveira@sydney.edu.au (P.A.S.); 3 Adrenal Steroids Laboratory, ANZAC Research Institute, University of Sydney, Sydney 2139, Australia; ktsa5143@uni.sydney.edu.au (K.H.-Y.T.); mark.cooper@sydney.edu.au (M.S.C.); 4 Bone Biology Group, ANZAC Research Institute, University of Sydney, Sydney 2139, Australia; sarah.kim1@sydney.edu.au; 5 Burns Unit, Concord Repatriation General Hospital, Sydney 2139, Australia; 6 Andrology Laboratory, ANZAC Research Institute, University of Sydney, Sydney 2139, Australia; simanainenu@gmail.com (U.S.); djh@anzac.edu.au (D.J.H.)

**Keywords:** major burn injury, androgens, dihydrotestosterone, wound healing, inflammation, mouse model

## Abstract

Androgens have been known to inhibit cutaneous wound healing in men and male mice. However, in children with major burn injuries, a synthetic androgen was reported clinically to improve wound healing. The aim of this study is to investigate the role of dihydrotestosterone (DHT) as a new therapeutic approach in treating major burn injury. In the present study, mice received systemic androgen treatment post major burn injury. Wound healing rate and body weight were monitored over 21 days. The serum level of inflammatory cytokines/chemokines were measured using multiplex immunoassays. In addition, splenocyte enumeration was performed by flow cytometry. Healing phases of inflammation, re-epithelialization, cell proliferation and collagen deposition were also examined. In results, DHT treated mice lost less weight and displayed accelerated wound healing but has no impact on hypermetabolism. Mice, after burn injury, displayed acute systemic inflammatory responses over 21 days. DHT treatment shortened the systemic inflammatory response with reduced splenic weight and monocyte numbers on day 14 and 21. DHT treatment also reduced wound infiltrating macrophage numbers. In conclusion, DHT treatment facilitates local wound healing by accelerating the resolution of inflammation, but not through alterations of post-burn hypermetabolic response.

## 1. Introduction

Both cutaneous (non-burn) and burn injuries immediately trigger the local wound healing process. This involves the four complex and overlapping phases of haemostasis, inflammation, proliferation and remodelling [1,2]. In contrast to simple cutaneous wounds, major burn injury simultaneously also triggers a systemic pathophysiological stress response and hypermetabolism [3]. The systemic hypermetabolic response refers to a significant increase in the resting energy expenditure, which is largely driven by elevated levels of circulating catecholamines, glucocorticoids and pro-inflammatory cytokines following burn injury [4]. A greater than 20% total body surface area (TBSA) full-thickness burn injury stimulates hypermetabolism in humans [3]. Furthermore, hypermetabolic burn patients also suffer from endocrine dysfunction, immune compromise, insulin resistance and whole-body catabolism [5,6]. These clinical features are associated with delayed recovery, prolonged hospital admission and increased morbidity and mortality. Currently available treatments to ameliorate hypermetabolism in major burn patients include local measures to reduce systemic cytokine stimulus (early excision, closure of the wound) as well as nutritional support and pharmacologic modalities such as androgens (testosterone, oxandrolone) and other anabolic hormones (insulin, growth hormone etc.) [4].

In male mammals, the major circulating androgen is testosterone, which is secreted by testicular Leydig cells. After testicular secretion, a small proportion of circulating testosterone (5~10%) is converted to the more potent androgen, dihydrotestosterone (DHT) by 5α-reductase enzymes [7,8]. In previous studies, castration of male mice to reduce circulating androgens, accelerates cutaneous wound healing through the attenuation of the local inflammatory response and increased extracellular matrix (ECM) deposition [9,10,11]. The castration effect on wound healing was counteracted by the restoring androgens in orchiectomised mice [9,10,11]. Delayed wound healing was also reported after chemical blockade of testosterone conversion to DHT or in genetic androgen receptor (AR) knockout mice [9,11,12,13]. In these studies, androgens play an inhibitory role in cutaneous wound healing through the increased production of IL-6 and TNF and reduced wound re-epithelialization and collagen deposition [9,11,12,13].

Systemic inflammation is crucial in wound healing by supplying the growth factors, cytokines and chemokines needed to recruit immune cells mainly neutrophil, monocytes and macrophages to the site of burn injury. In cutaneous wounds, the systemic and local inflammatory phase lasts for 5–7 days. However, a major burn may display chronic persistent inflammation with a marked release of abundant pro-inflammatory mediators that linked to inflammatory response syndrome, sepsis, and multi-organ failure in patients.

In contrast to the known inhibitory effects of androgens by prolonging inflammation in cutaneous wound healing, clinical studies have reported that testosterone and the synthetic androgen, oxandrolone can enhance recovery from a burn injury. Oxandrolone-treated burn patients maintain body mass better and have improved body composition and hepatic protein synthesis during the acute post-burn phase [14,15,16,17,18]. Although there are reports of improved patient outcomes mainly in treated children (with low circulating androgens) after major burns, the role of androgens in major burn wound healing processes is still unknown.

DHT is the most potent natural androgen, with higher affinity and slower dissociation from AR binding leading to greater molar potency in the transactivation of the AR [8]. Most importantly, DHT lacks the hepatotoxicity of 17α–alkylated synthetic androgens like oxandrolone, highlighting its overlooked potential as an important therapeutic option for male burn patients. Therefore, we hypothesise that pure androgen, DHT, can promote major burn wound healing via altering post-burn inflammation response. This study aims to investigate DHT treatment as a new therapeutic approach in treating major burn injury.

## 2. Results

### 2.1. DHT Accelerates Local Wound Healing in Mice Post Major Burn Injury

**Figure 1:**
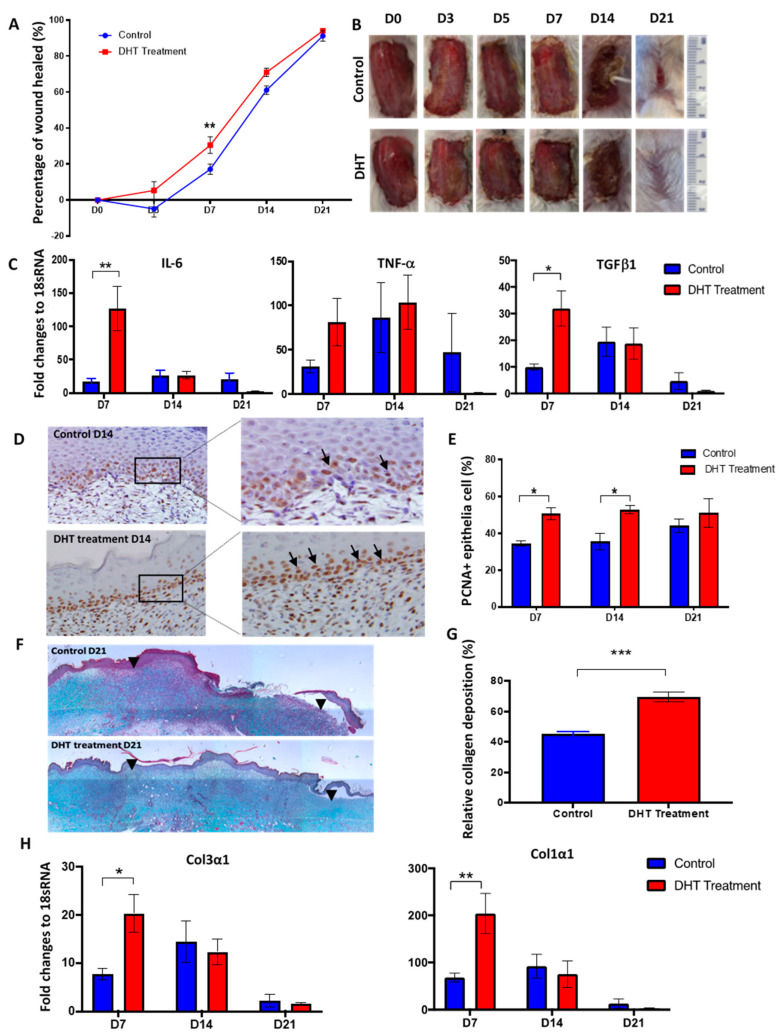
Major burn wound healing is accelerated in dihydrotestosterone (DHT)-treated mice. (**A**) Wound healing rates were quantified as the percentage of the wound healed. (**B**) Progression of burn wound healing, with healing completed by day 21 in DHT-treated mice. (**C**) Fold changes relative to housekeeping gene 18sRNA for IL-6, TNFα, TGFβ mRNA expression in the wound in control and DHT treatment. (**D**) Represented images showing more proliferating cell nuclear antigen (PCNA) staining in DHT-treated mice on day 14. (**E**) Quantification of positive PCNA^+^ epithelia cells per 30 cells by PCNA staining, illustrating a significant increase of proliferating epithelia cells in DHT-treated mice at D7 and day 14. (**F**) Masson trichrome staining showing more collagen deposition in the wound of DHT treatment group. Black arrowheads indicate edges of the wound. (**G**) Quantification of collagen deposition during the late phase (day 21) of wound healing. (**H**) Col3α1 and Col1α1 mRNA expression relative to 18sRNA in the wound in control and DHT treatment. *n* = 6 per time point, * *p* < 0.05, ** *p* < 0.01, *** *p* < 0.001. Statistical significance was analysed using repeat measurement on two-way ANOVA (**A**) and two-way ANOVA (**C**–**H**).

We investigated the effects of DHT on a major burn injury throughout the wound healing process. In the DHT-treated male mice, accelerated wound healing was observed at the early time points (Figure 1A,B). On day 7, the wound of DHT-treated mice had healed by 30.5 ± 12.1%, whilst only 7.1 ± 7.5% of the wound was healed in the control mice (Figure 1A). Elevated mRNA expression of IL-6, TNF-α and TGF-β1 were detected at the wound of DHT-treated mice on day 7 when compared to the control mice (Figure 1C). In addition, the number of proliferating cells in the basal layer of the epidermis was significantly increased in the DHT group on day 7 and day 14, with average 50.7 ± 2.2% and 52.8 ± 2.1% positive staining of proliferating cell nuclear antigen (PCNA) cells compared to 34.4 ± 1.4% and 35.6 ± 4.5% in the control group (Figure 1D,E). These findings suggest that DHT treatment can promote cell proliferation that may contribute to enhancing local wound healing. However, no difference in re-epithelization between the two groups was noted suggesting that DHT treatment did not affect keratinocyte migration (Supplementary Materials Figure S2).

Masson’s trichrome staining of wounds was used to assess differences in tissue remodelling between the two groups. A significantly higher relative collagen fibre percentage was found in the wound site of DHT-treated mice compared to control mice on day 21 (Figure 1F,G). These results are consistent with the increased mRNA expression of immature collagen type III and mature collagen type I in the wounds of DHT-treated mice at day 7. The mRNA expression of collagen type III and collagen type I increased by approximately 20-fold and 205-fold, respectively in the DHT-treated group on day 7 compared to the 8-fold and 68-fold increase observed in the control group, respectively (Figure 1H). Histological analysis of wound tissues supports the role of DHT in accelerating local wound healing post major burn injury through enhancing basal cell proliferation and collagen deposition.

### 2.2. DHT Better Maintained Body Weight but Had No Effects on Metabolic Response Following Major Burn Injury

**Figure 2:**
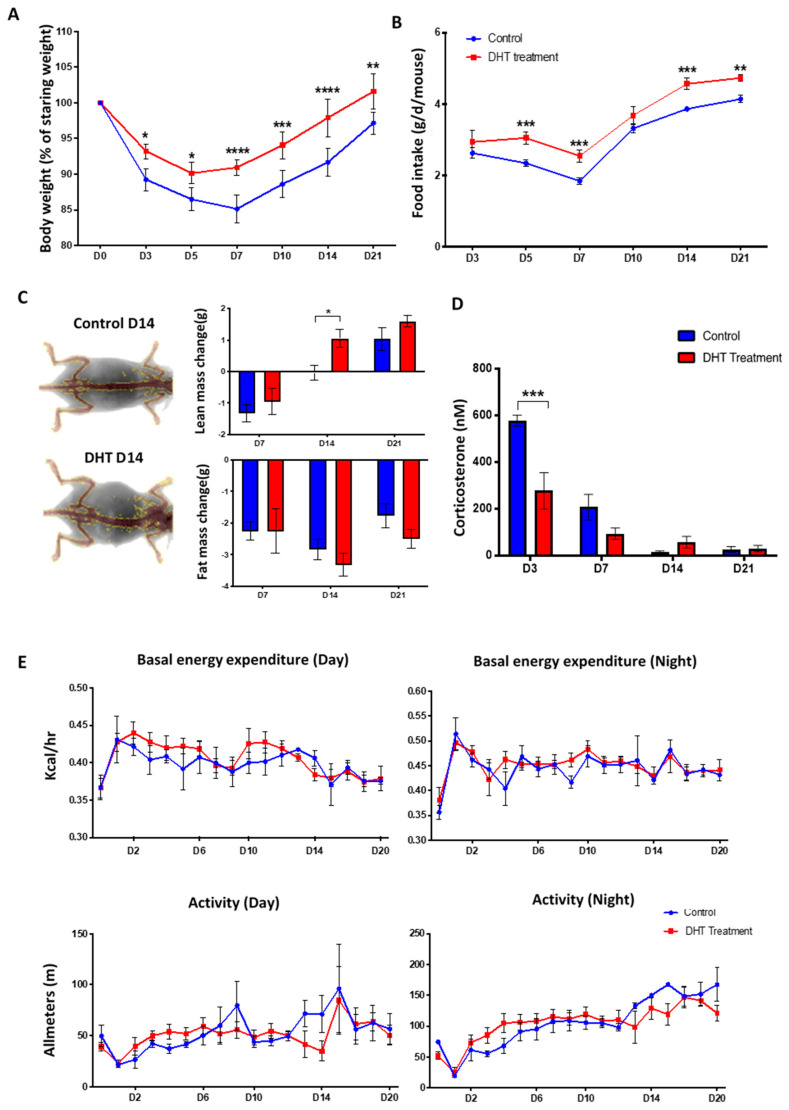
General catabolic response to major burn injury.(**A**) Bodyweight (% of starting weight post-burn injury) showing reduced weight loss in DHT treatment group. (**B**) Food intake in control and DHT treatment mice throughout the wound healing process. (**C**) Lean and fat mass change from baseline taken prior to burn injury, as assessed by dual-energy X-ray absorptiometry (DEXA), showing increased lean mass weight at day 7 after DHT treatment. (**D**) Significant lower serum corticosterone concentration was observed in the DHT treatment group on day 3 post-burn when compared to control. (**E**) No significant differences were observed from both basal energy expenditure and activity of the mice recorded during the day and night from metabolic cage throughout the wound healing process, control and DHT treatment. *n* = 6 per time point, * *p* < 0.05, ** *p* < 0.01, *** *p* < 0.001, **** *p* < 0.0001. Statistical significance was analysed using repeat measurement on two-way ANOVA (**A**,**B**,**E**) and two-way ANOVA (**C**,**D**).

Major burn injury induces hypermetabolic stress responses in both human [6] and mice [19]. Clinical features of hypermetabolism include significant weight loss, increased levels of stress hormones, and elevated energy expenditure [4]. These features were assessed in both experimental groups to investigate the effect of DHT treatment on the hypermetabolic response to burn injury. Over the 21 days of wound healing, DHT-treated mice showed significantly better maintained body weight and higher food intake compared to the control group (Figure 2A,B). Only 7–9% of body weight loss was noted in DHT-treated mice on days 3 and 5, respectively, that was followed by rapid weight gain from day 7, onwards. However, in the control group, animals lost 14.9 ±1.9% of body weight on day 7, followed by significantly slower weight gain over the 21 days.

Changes in body composition were assessed using a dual-energy X-ray absorptiometry (DEXA) that measured total lean mass and fat mass of the animals. Both groups of mice showed lean mass loss initially on day 7, but a significant recovery was observed in DHT-treated group (1.1 ± 0.3 g) when compared to control (−0.03 ± 0.2 g) on day 14 (Figure 2C). No difference was observed in the change of fat mass between these two groups over the experimental period (Figure 2C). Systemic corticosterone levels in the serum of mice were measured to assess stress response, with DHT-treated mice demonstrating significantly lower level at 278.1 ± 156.5 nM compared to the control group 578.8 ± 47.2 nM on day 3, post debridement by ELISA (Figure 2D).

Post-burn injury, all mice experienced an increase in energy expenditure and reduced movement. However, DHT showed no effect on either energy expenditure or movement of the mice post major burn injury in either day or night time (Figure 2E). Average basal energy expenditure in daytime for DHT-treated mice was measured at an average of 0.42 kcal/h, which peaked on day 1 compared to 0.43 kcal/h in the control group; while at nighttime, the average basal energy expenditure increased to an average of 0.51kcal/h in DHT mice and 0.49 kcal/h in control mice, respectively. The reduced movement was immediately observed in both groups on day 1 post-burn injury with movement down to approximate 21.77 m in the daytime and 19.73 m at night (Figure 2E). Both groups showed a similar pattern of movements, which increased gradually over 21 days in parallel with recovery and wound healing.

### 2.3. DHT Treatment Enhances Inflammatory Cytokines and Chemokines Release

**Figure 3:**
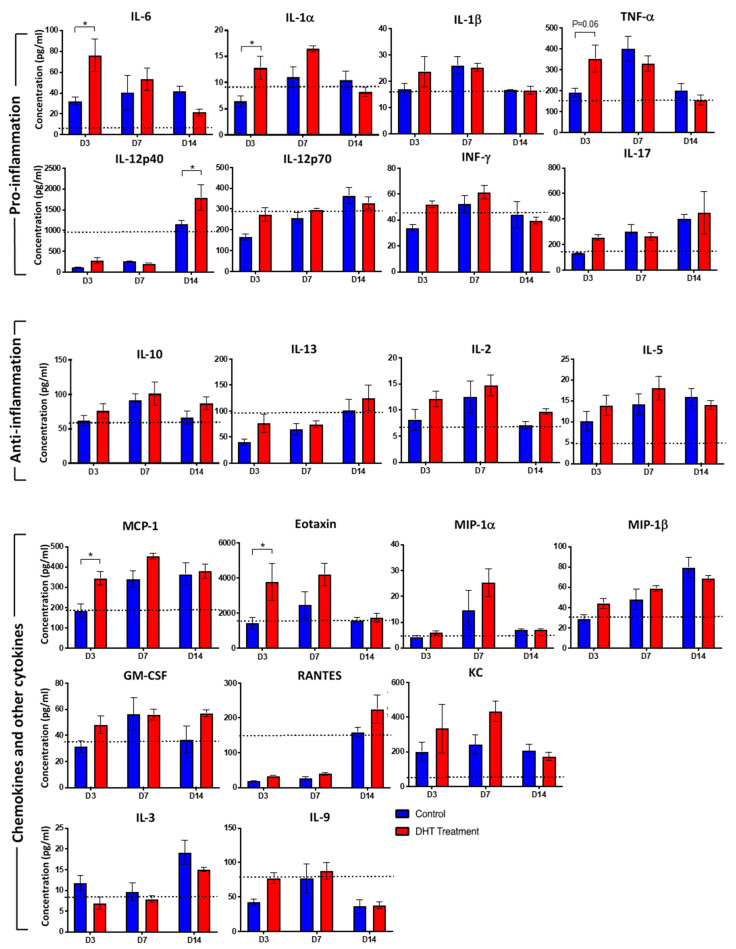
Cytokines response to major burn injury.Mouse cytokine 23-plex assay showing inflammatory cytokines regulating the systemic inflammatory response to control (Blue) and DHT treatment (Red) post major burn injury. IL-1α, IL-6, MCP-1, Eotaxin raised in DHT-treated mice at day 3 and IL-12p40 raised in DHT-treated mice at day14. (IL-4 and G-CSF not shown as the concentration were below detectable levels). Data presented as mean with a standard error of the mean (SEM). Black dotted lines indicate cytokine levels in non-injured control mice. *n* = 3 per time point for control and DHT treatment. * *p* ≤ 0.05 DHT treatment vs. control.

Inflammatory response involving elevated cytokines and growth factors to protect against the risk of infection is fundamental to the burn wound healing process. Among twenty-three inflammatory cytokines and chemokines analysed, IL-6 was significantly increased to 76.2 pg/mL in DHT treated mice, compared to 32.3 pg/mL in control mice on day 3, while in the non-burn mice, 21.5 pg/mL were measured (Figure 3). Thereafter, the level of IL-6 dropped sharply down to 21.4 pg/mL in the DHT treated mice on day 14 but still remained high in the control mice at 41.9 pg/mL. Similarly, IL-1α and TNF-α were significantly increased in DHT-treated mice (12.8 pg/mL, 354.1 pg/mL) on day 3 when compared to control mice (6.4 pg/mL, 191.7 pg/mL), suggesting a greater inflammatory response in the DHT-treated mice early post-injury. IL-12 (p40) was also significantly increased at 1795.6 pg/mL in DHT treated mice, compared to 1149.0 pg/mL in control mice which is similar to 1010.0 pg/mL in non-burn mice at day 14 (Figure 3). Other significantly increased chemokines included MCP-1 and Eotaxin (CCL11), which were both raised on day 3 in DHT treated mice (344.5 pg/mL, 3768.7 pg/mL), compared to control mice (184.7 pg/mL, 1433.7 pg/mL) and non-burn mice (188.3 pg/mL, 1495.0 pg/mL) (Figure 3).

### 2.4. DHT Treatment Preserves Normal Splenic Structure Post Major Burn Injury

**Figure 4:**
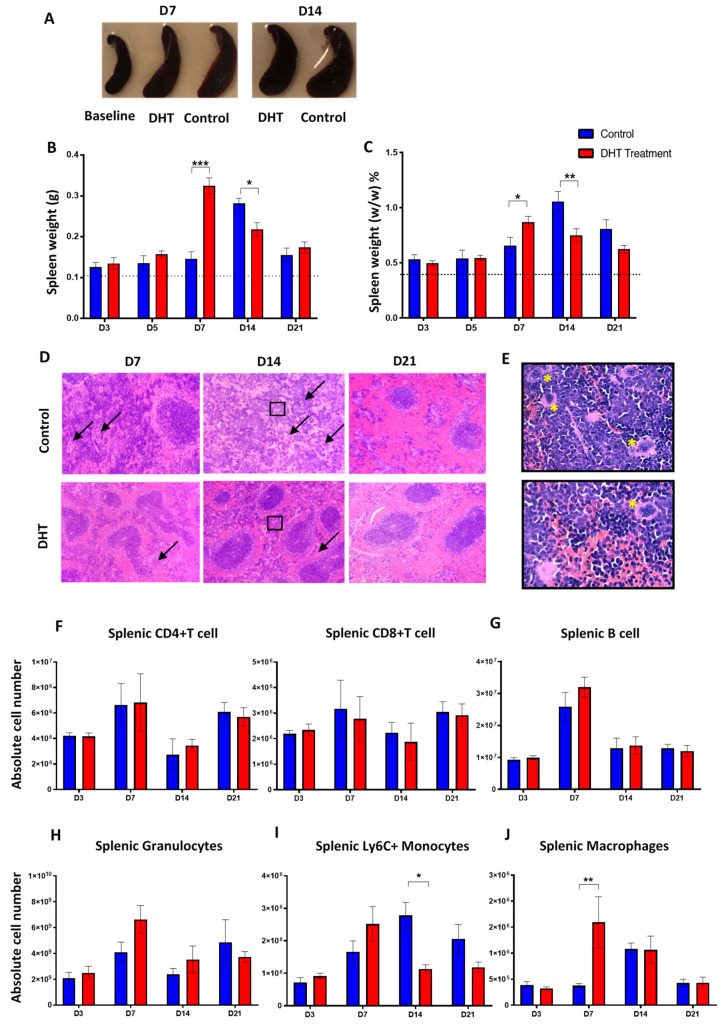
Spleen changes post major burn injury.(**A**) Spleen enlargement in both groups post-major burn injury when compared to the baseline (non-injury) mice. (**B**,**C**) Spleen weight (g) and (%, *w*/*w*) increased significantly in control mice at day 14 while spleen weight (g and %) increase significantly in DHT-treated mice at day 7. (**D**) Representative H&E staining showing disorganised white pulp area with extensive infiltration of multi-nuclei cells (black arrow) in the red pulp area (**E**). (**F**–**H**) Flow cytometry to identify the numbers of splenic T cell (CD4^+^/CD8^+^, IAIE^+^, Lin^+^), B cell (IAIE^−^, Lin^−^) and granulocytes, which all peaked on day 7, followed by a reduction in both groups. (**I**,**J**) Flow cytometry to identify the numbers of splenic monocytes (Ly6C^+^, Lin^−^) and splenic macrophages (F4/80^+^, Lin^−^). Early increase of Ly6C+ monocytes, followed by a significant increase of macrophages on day 7 was observed in DHT treatment when compared to control. *n* = 6 per time point for control and DHT-treated mice. * *p* < 0.05, ** *p* < 0.01, *** *p* < 0.001.

In the present study, splenomegaly was observed in all animal post-burn injury (Figure 4A) which is correlated with the increased systemic inflammation profile. In healthy non-injured mice, the weight of spleen was measured at an average of 0.1g (approximately 0.39%) of total body weight. Post major burn injury, the weight of the spleen in control mice was increased on day 14 (0.3 ± 0.03 g, 1.1 ± 0.2% *w*/*w*) then reduced to 0.2 ± 0.03 g (0.8 ± 0.2% *w*/*w*) on day 21 (Figure 4B,C). In the DHT treatment group, mice had the greatest spleen weight on day 7 (0.3 ± 0.05 g, 0.9 ± 0.1% *w*/*w*) which was 7 days earlier than the control group (Figure 4B,C). Histological analysis demonstrated that all control mice had an abnormal splenic structure with disrupted compartmentations post-injury over 21 days (Figure 4D). Moderately to extensively disorganised white pulp with evident but poorly discernible or indistinct regions were observed on both day 7 and day 14 (Figure 4D). The follicular structure was barely distinct from red pulps and massive infiltration of immune cells (granulocytes) was also found (Figure 4E). In contrast, with DHT treatment, the histological images of spleens showed a mild structural disruption on day 7, but recovery of white pulps can be observed as early as day 14 and on day 21 (FigFigure 4D).

There were no significant differences found in other organ weight% (*w*/*w*) including non-androgen dependent tissues (liver, brown adipose tissue and white adipose tissue, Supplementary Materials Figure S3A) and androgen-dependent tissues (testes, kidneys and seminal vesicle dry, Supplementary Materials Figure S3B). Only the seminal vesicle (wet weight) was significantly higher in the DHT-treated group at day 14, suggesting minimal androgen effect from DHT treatment.

### 2.5. Monocytes and Macrophages are Critical for Facilitating Major Burn Wound Healing

Since the infiltration of immune cells in the spleen was observed, we further investigated the role of DHT in regulating immune cell populations. The number of different blood and splenic immune populations were enumerated using flow cytometry. In both groups of mice, the absolute number of CD4^+^ and CD8^+^ T cell, B cell and granulocytes in spleens all peaked on day 7, followed by a reduction (Figure 4F–H). The number of Ly6C^+^ splenic monocytes in control mice was significantly higher than the DHT-treated mice on day 14 (Figure 4I). Although the numbers of Ly6C^+^ monocytes reduced by day 21, the monocytes in the control group remained higher compared to the DHT-treated group. The peak of Ly6C^+^ monocytes number in the spleens of DHT-treated mice was observed on day 7 with an increased population followed by a reduction observed on day 14 and day 21 (Figure 4I). The increase in the number of monocytes on day 7 in DHT-treated mice appears correlated with the higher production of inflammatory cytokines further confirmed a more turnover of immune response after DHT treatment.

**Figure 5:**
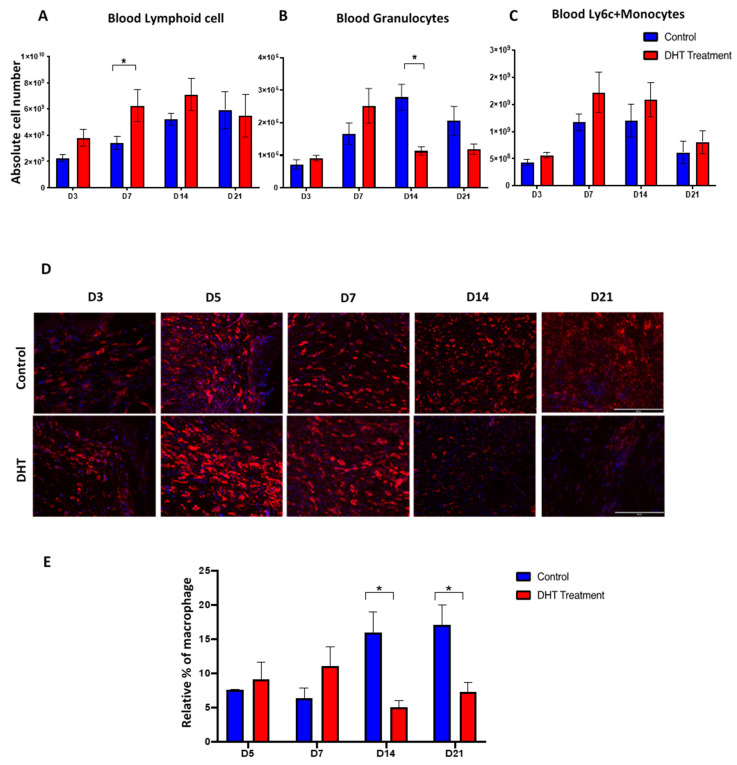
Blood immune populations and wound macrophage changes post-major burn injury. (**A**–**C**) Flow cytometry to identify the numbers of lymphoid cell, granulocytes and Ly6C^+^ monocytes in the blood. *n* = 6 per timepoint. * *p* < 0.05 (**D**) Immunostaining of F4/80 labelled macrophage at the wound site. Early increase in F4/80 macrophage was observed in DHT treatment group at D5 and D7. (**E**) quantification of relative F4/80 macrophage percentages showing a late increase in F4/80 macrophage number in the control group. *n* = 4 per time point * *p* < 0.05.

The number of splenic macrophages was also significantly increased in the DHT treatment group on day 7 (Figure 4J). In comparison, the splenic macrophages in the control group reached its peak population 7 days later, on day 14 (Figure 4J). DHT-treated mice had elevated level of blood Ly6C^+^monocytes on days 7 and 14, corresponding to the high Ly6C^+^ monocytes observed in the spleen (Figure 5C). In addition, DHT-treated had raised blood lymphocytes number on day 3, compared with control mice (Figure 5A). While control mice had significant increases of granulocyte numbers on day 7 and 14, DHT-treated mice peaked at day 7 but dropped sharply thereafter (Figure 5B). To correlate our observations in spleen and wound tissue, we performed histological staining using anti-F4/80 antibodies to identify macrophages in the wound area. The number of macrophages gradually increased in the wound area from day 3 to 21 in the control group. However, in DHT-treated mice, an increased number of macrophages was observed on day 5 and 7 followed by significant reduction on day 14 and day 21 (5% wound macrophage versus 15% of that in the control group at day 14 and 8% versus 18% in the control group at day 21, *p* < 0.05, Figure 5D,E).

## 3. Discussion 

In this study, we examined the role of a pure (non-aromatisable) androgen, DHT, in the local wound healing process and the systemic hypermetabolic response following major burn injury in mice. Our results showed that DHT has a positive impact on both local wound healing and metabolic catabolic responses, which differ from those reported after cutaneous injury.

DHT-treated mice showed a faster healing rate, particularly in the early stages (day 3–14) of the healing process. DHT contributed to these effects by influencing local inflammation, cell proliferation of keratinocytes and ECM production. DHT-treated mice also exhibited a favourable local inflammatory response in the wound area demonstrated by an early increase of pro-inflammation cytokines IL-6 and TNF post-burn on day 7 followed by a rapid reduction on day 14 and 21, which counteracts the elevated level of anti-inflammation cytokine TGFβ-1 (Figure 1C). Our findings are in contrast with previous studies that show that androgens inhibit cutaneous wound healing by enhancing TNF production and prolonging inflammation following non-penetrating skin injury [13]. Moreover, burn injury wounds in the DHT treatment group exhibited more connective tissues and collagen production over the remodelling phase. This also contrasts with previous findings that androgens suppress collagen production but contribute to collagen degradation in cutaneous non-burn wound healing [9,12,13]. The different effects of DHT on local wound healing of non-burn cutaneous and major burn injury may be explained by the effect of DHT on systemic responses following burn injury. Major burn injury simultaneously induces a systemic hypermetabolic response in humans [4] and mice [19], resulting in a pronounced and prolonged increase in basal energy expenditure (BEE), a greater stress response with higher levels of corticosterone (in mice) and cortisol (in humans) as well as inflammatory cytokines all culminating in profound catabolic consequences [19].

In severely burned male patients, testicular steroid production is substantially decreased. The reduction in circulating testosterone level contributes greatly to the loss of lean body mass observed in burned patients [20,21,22]. Analogous findings were observed in control burn-injured mice in the current study (Figure 2A). DHT-treated mice maintained a more stable body weight following injury, with increased lean body mass compared to mice in the control group. The faster recovery of body weight was associated with increased food intake (Figure 2B). This finding is supported by clinical data that the administration of synthetic 17-α alkylated androgen, oxandrolone, can improve gross parameter of lean body mass, total body mass, bone mineral composition, strength and shorten the length of hospital stay for major burn injury child patents [4,15,16,18]. Moreover, serum level of corticosterone on days 3 and 7 post-injury was low in the DHT group (Figure 2D), indicating that DHT can minimise stress response, perhaps contributing to better food intake and body weight recovery as acute stress response has been reported to suppress appetite and food intake [23,24]. However, DHT treatment had no effect on basal energy expenditure or movement in burn-injured mice, although DHT is an anabolic hormone that can increase protein synthesis and ameliorate catabolism post burn injury. Similar findings were reported before in clinical studies in which oxandrolone showed no effect on resting expenditure rate or basal metabolic rate in children with burn injuries [18].

In this study, we have demonstrated an overall increase in inflammatory cytokines and chemokines post-burn injury (Figure 3) as previously reported clinically [25,26,27,28]. Significantly increased pro-inflammation cytokines IL-1α, IL-6 and TNF on day 3 in DHT mice (Figure 1C and Figure 3) suggested that DHT treatment may play a role in accelerating the turnover of early inflammatory response. While the elevated serum level of the macrophage recruiting cytokines such as IL-12(p40), chemokine RANTES at a later stage of wound healing (Figure 3) indicating early resolution of inflammation which correlates to the increased cell proliferation and collagen deposition observed.

Splenomegaly after major burn injury in both experimental groups was observed, confirming a burn injury-induced systemic inflammation change (Figure 4A). Enumeration of splenic immune populations demonstrated an increase of granulocytes, monocytes and macrophages in DHT-treated mice 7 days earlier than control animals (Figure 4H–J). These findings suggested that DHT enhanced systemic inflammation post major injury, resulting in a quick resolution of inflammation. Unlike cutaneous wound healing, during which androgens prolong inflammation phase [9,11,13,29], our study reported after DHT treatment, the majority of inflammation markers and immune cell numbers dropped to normal level after 14–21 days post major burn injury.

In major burn wound healing, monocytes circulate through the blood-stream and extravasate into wound site during inflammation phase. In addition to the bone marrow, splenic monocytes produced within the subcapsular red pulp can also be recruited to wound site [30,31]. This recruitment of pro-inflammatory monocyte is predominantly dependent on MCP-1 and its receptor CCR2 [31]. A recent study found that circulating monocytes expressing a high level of Ly6C^+^ infiltrate into the skin can differentiate into wound repair macrophages expressing F4/80 antigen [30,31]. These macrophages contribute to phagocytosing dead tissues, stimulating angiogenesis, promoting granulation tissue formation, secreting extracellular matrix and re-epithelization the wound [30,31,32]. In our study, an elevated circulating MCP-1 concentration was observed in DHT-treated mice on day 3 (Figure 3), resulting more Ly6C^+^ monocytes being mobilised into the circulation and recruited to the spleen (Figure 4H). This finding correlates with the increased in macrophages in the wound area of the DHT-treated mice (Figure 5) that facilitate wound healing by enhancing cell proliferation, re-epithelialization and collagen deposition as suggested by previous studies [33,34].

**Figure 6:**
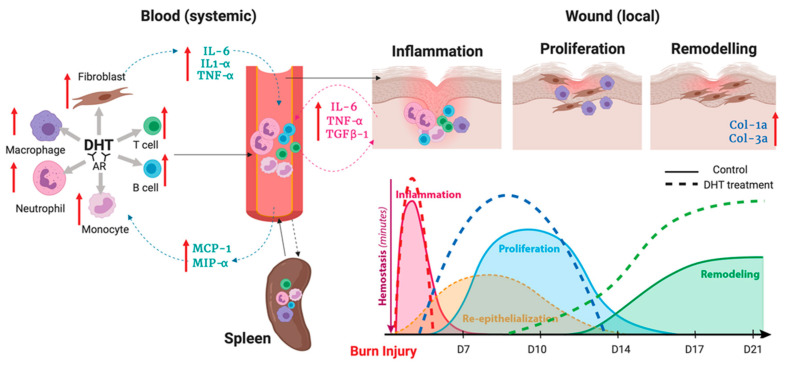
Illustration of DHT enhances major burn injury wound healing via accelerating inflammation turnover, resulting in a fast resolution of inflammation phase followed by early proliferation and remodelling.

Taken together, our findings from the current study shows that burn injuries induced hypermetabolic-catabolic and stress response and resulted in the production of pro-inflammation cytokines including IL-1α, IL-6 and TNF. Thereby DHT acting via AR on neutrophil, T cell, B cell, monocytes and macrophages are able to recruit other immune cells into the circulation via elevated inflammation factors. Circulating immune cells then infiltrate and recruit to the spleen. The increase in MCP-1 level at the wound area and systemically recruits more monocytes to the blood circulation and the wound site. These monocytes are then differentiated to macrophages that are involved in removing bacteria, preventing infection and contributing in collagen disposition once the inflammation is resolved with the increase of anti-inflammation cytokine such as TGFβ-1 (Figure 6). We show here that DHT treatment induced acceleration of the inflammatory turnover both locally and systemically as the key in promoting major burn wound healing. DHT, a potent natural pure androgen, showed no adverse effects in mice over 21 days of treatment, highlighting its potential as a novel therapeutic approach for treating a major injury.

This research is comprehensive in providing evidence on androgen treatments in major burn wound healing. However, a few limitations of these studies need to be acknowledged. Firstly, burn wound healing studies were conducted only in male mice, and differences in the response to the androgen treatments may exist in female mice. Secondly, due to differences between mouse and human metabolism, wound healing mechanism and skin structure, translational research on the effect of systemic DHT administration needs to be conducted in the clinical settings of a major burn injury. The findings will be valuable to further evaluate its therapeutic potential.

## 4. Materials and Methods

### 4.1. Major Burn Injury Model and Wound Healing Experiment

Male BALB/c mice (Animal Resource Centre, Murdoch, WA, Australia) (*n* = 120) were housed with free access to water, in the specific-pathogen-free Translational Research Facility (TRF) at the ANZAC Research Institute. The environment was closely controlled at 24–26 °C and 44–46% humidity under a 12:12 h light–dark cycle with lights on at 6 am. All protocols were approved by the Sydney Local Health District Animal Welfare Committee (Protocol No. 2018/020) under the Australian National Health and Medical Research Council Guidelines for animal experimentation.

At 12 weeks of age, mice weighted ~22–27 g were anaesthetised with 3% isoflurane and the dorsum of the mouse was shaved. In the treatment group, mice were subdermally implanted with 1 cm silastic tube filled with ~10mg crystalline DHT [35] in the neck region, while mice in the control group were also implanted with an empty silastic tube.

At the same day of DHT implantation, mice were then subjected to a major burn injury as previously described [19]. Briefly, a 4 cm^2^ contact burn wound (representing ~10% TBSA) was generated using a hot brass rod. The wound site was debrided 48 h later, removing the damaged skin and to avoid infection, prior to wound dressing for 10 days. Wound tissues were harvested at day 3, 5, 7, 14, 21 post debridement (day 0). Wound samples were bisected for histology and immediately snap-frozen in liquid nitrogen for molecular analysis. Androgen sensitive organs (kidney, testes, seminal vesicle), liver, brown adipose tissue, and white adipose tissue were also collected for further analysis. The spleen was weighed and harvested for analysis by flow cytometry. Wound healing rate was quantified using the Visitrack digital instrument (Smith and Nephew).

### 4.2. Body Composition and Food Intake

Bodyweight and food intake were measured before and after burn injury at days 3, 5, 7, 10, 14, 21. Body compositions were measured using dual-energy X-ray absorptiometry (DEXA, Lunar Piximus, GE Lunar Corp, WI, USA) following the manufacturer’s manual.

### 4.3. Metabolism

Gas exchange, energy expenditure and movement of mice (*n* = 4 per group) were individually continuously measured following major burn injury in Promethion metabolic cage systems for 21 days (Sable Systems International, North Las Vegas, NV, USA). Energy expenditure was calculated using the Weir equation: Kcal/h = 60 × (0.003941 × VO_2_ + 0.001106 × VCO_2_). Results were analyzed by R and R studio software.

### 4.4. Histology, Immunocytochemistry, and Image Analysis

Wound tissues were embedded in paraffin. Multiple 5 µm sections were stained with hematoxylin and eosin (H&E) for general histological analysis; Masson’s Trichrome for collagen deposition; proliferating cell nuclear antigen (PCNA) staining for cell proliferating. The total number of positive stained cells/30 cells in 3 random chosen wound areas was counted and examined by two individual researchers. Multiple sections were stained with anti-mouse F4/80–Alexa Fluor (AF) 647 (1:100, BM8; BD Biosciences, NSW, Australia) for detecting macrophage at wound site.

### 4.5. Real-Time Quantitative Polymerase Chain Reaction

Total RNA from normal skin and wound tissues were extracted with TRI Reagent (Sigma Aldrich) according to the manufacturer’s instructions. Polymerase chain reaction (PCR) primers for IL-6, TNF-α, TGF-β1, Col3α, Col1α1, (Supplementary Materials Table S1) were designed using Primer-BLAST Software. RT-qPCR was performed using Ssoadvanced Universal SYBR Green Supermix (Bio-Rad, Hercules, CA, USA). The CFX Manager Software was used to obtain cycle threshold (Ct) values for each sample, which were expressed as a fold change relative to expression of 18s RNA housekeeping gene.

### 4.6. Obtaining Single-Cell Suspensions from Mouse Tissues

Peripheral blood was obtained from mice by tail bleeding. Single-cell suspensions were made from mouse spleen by mechanical disruption through 70-μm cell strainers (Edwards, NSW, Australia) in RPMI 1640 media supplemented with 2% heat-inactivated fetal bovine serum (Thermo Fisher, WI, USA; 2% FCS/RPMI 1640). Red blood cells were removed from spleens with 1 × RC lysis buffer (eBioscience, CA, USA). To enumerate, cells were counted in a fixed volume (10–50 μL) using the ACCURI C6 flow cytometer (BD Biosciences) for spleen samples or XN-450 automated haematology analyzer (Sysmex, NSW, Australia) for peripheral blood.

### 4.7. Flow Cytometry

Single-cell suspensions were stained in an FACS buffer (0.1% BSA and 2 mM EDTA in PBS (Sigma Aldrich)) using optimised concentrations of Fc Block (2.4G2; BD Biosciences) and then combinations of the following fluorochrome-conjugated anti-mouse monoclonal antibodies (mAbs) for peripheral blood samples: IA-IE (M5/114-15.2) from BD Biosciences, Ly6C (HK1.4) from eBioscience and F4/80 (BM8) from BioLegend (NSW, Australia); for spleen samples: CD11c (N418), CD4 (GK1.5), B220 (RA3-6B2), CD8a (53-6.7), F4/80 (BM8), IA-IE (M5/114-15.2), Ly6C (HK1.4). To assist in identifying rare myeloid populations, cells were also stained with a lineage (Lin) mixture containing biotinylated mAbs: CD3e (145-2C11), CD19 (1D3) from BD Biosciences, and Ly6G (1A8) and NK1.1 (PK136) from BioLegend. Lin mAbs were detected using streptavidin–Brilliant Violet 421 (BD Biosciences) (Supplementary Materials Figure S1). Data were collected on the ACCURI C6 flow cytometer (BD Biosciences) and analyzed using FlowJo software (Tree Star, version 9).

### 4.8. Cytokines Expression

The Bio-Plex Pro^TM^ Mouse Cytokine 23-plex Assay (Bio-Rad, Hercules, CA, USA) was used to profile the expression of 23 inflammatory mediators interleukin IL-1a, IL-1β, IL-2, IL-3, IL-4, IL-5, IL-6, IL-9, IL-10, IL-12p40, IL-12p70, IL-13, IL-17α, granulocyte colony-stimulating factor (G-CSF), granulocyte-macrophage colony-stimulating factor (GM-CSF), interferon-gamma (IFN-λ), monocyte chemoattractant protein-1 (MCP-1), macrophage inflammatory protein-1 alpha and beta (MIP-1α, MIP-1β) tumour necrosis factor-alpha (TNF-α), Chemokine ligand 5 (RANTES), eotaxin and chemokine ligand 1 (KC). Burn-injured and control mice had serum samples collected at day 3, 7 and 14 and measurements were performed according to the manufacturer’s instructions and analyzed using the Bio-Plex Manager Software (Bio-Rad, Hercules, CA, USA).

### 4.9. Corticosterone Assay

Blood sampling via tail snips was continuously collected in mice before burn injury and in the morning of day 3, 7, 14 and 21 post-debridement. All blood samples were centrifuged at 2000× *g* for 5 min at room temperature and stored at −80 °C until further analysis. Plasma corticosterone concentrations were measured using the DetectX^®^ Corticosterone Enzyme Immunoassay Kit (Arbor Assays, Ann Arbor, MI, USA).

### 4.10. Statistical Analysis

Repeated measurement analysis of variance (ANOVA) was used for all continuous variables including, wound healing rate, body weight changes and food intakes to correct bias caused by multiple observation from each mouse. The data are presented as mean ± SEM and significant differences were determined by two-way analysis of variance (ANOVA) and pair-T test with *p* ≤ 0.05 accepted for statistical significance. *n* = 6 per group per time point * *p* < 0.05, ** *p* < 0.01, *** *p* < 0.001.

This work was performed at the ANZAC Research Institute, Concord Hospital.

## Supplementary Materials

The following are available online at https://www.mdpi.com/1422-0067/21/17/6231/s1, Figure S1: Gating strategy for the identification of blood and splenic immune populations in DHT treated and control mice, Figure S2: Rate of re-epithelisation post burn injury, Figure S3: Androgen sensitive/non-sensitive organ weight, Table S1: List of primer sequence.

## Abbreviations

AR	androgen receptor
DHT	dihydrotestosterone
ECM	extracellular matrix
TBSA	total body surface area
PCNA	proliferating cell nuclear antigen
