# Supplementary material for: Dihydrotestosterone (DHT) Enhances Wound Healing of Major Burn Injury by Accelerating Resolution of Inflammation in Mice
Source: Int J Mol Sci. 2020 Aug 28;21(17):6231. doi: 10.3390/ijms21176231 (PMC7504698; doi:10.3390/ijms21176231)
Supplement: Supplementary file 1 [file ijms-21-06231-s001.pdf]

Supplementary Material

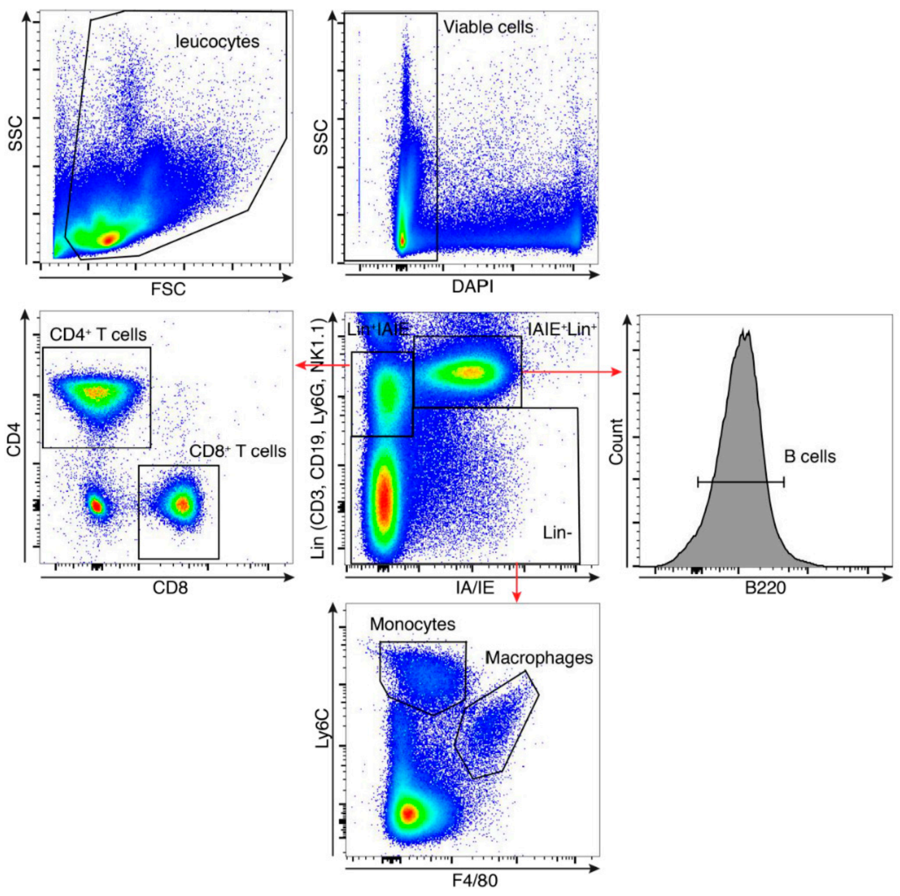

Supplemental figure 1. Gating strategy for the identification of blood and splenic immune populations in DHT treated and control mice. Representative dot plots illustrating the gating strategy used to identify the immune populations in the blood and spleens of experimental animals. The leucocytes were identified using forward and side scatter parameters and their viability was assessed by DAPI stain. The Lin$^+$ viable leucocyte population was then examined for their expression of B220 and IA/IE to identify B and T cells. T cell subsets were further characterised using CD4 and CD8. The remaining Lin$^-$ viable leucocytes were examined for their expression of F4/80 and Ly6C. Ly6C$^+$ cells were identified as monocytes while F4/80$^+$ cells were identified as macrophages.

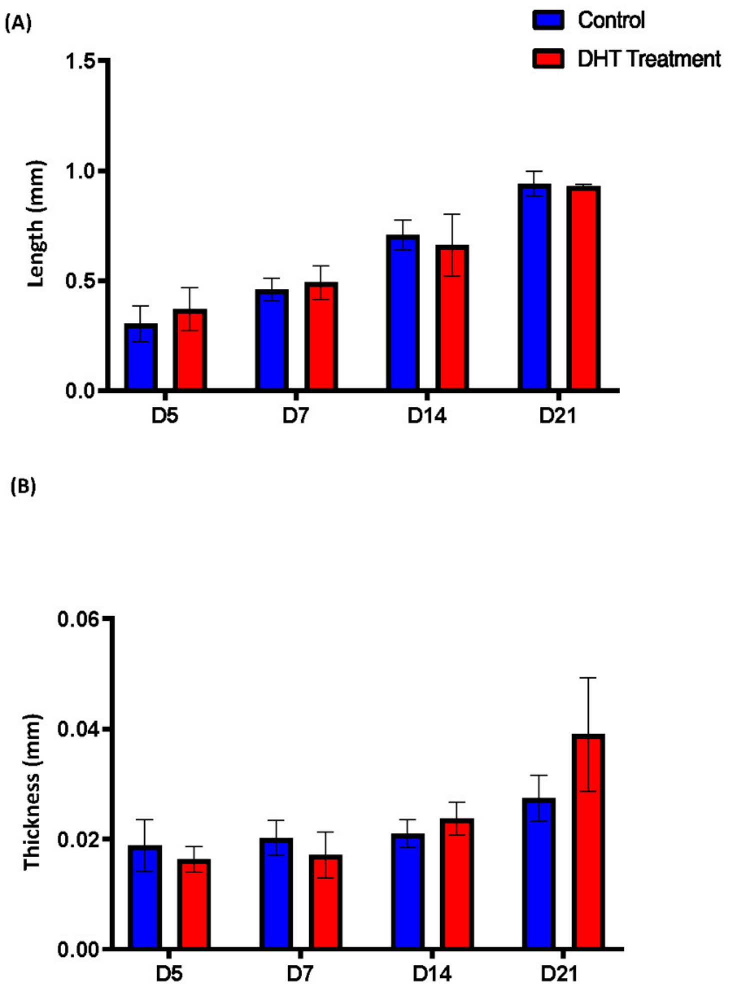

Supplemental figure 2. Rate of re-epithelisation post burn injury No difference was observed for the length and thickness of epithelium in DHT treated and control group at day 5, 7, 14 and 21 post burn injury, suggesting DHT treatment does not affect re-epithelialization. *N*=4 per time point.

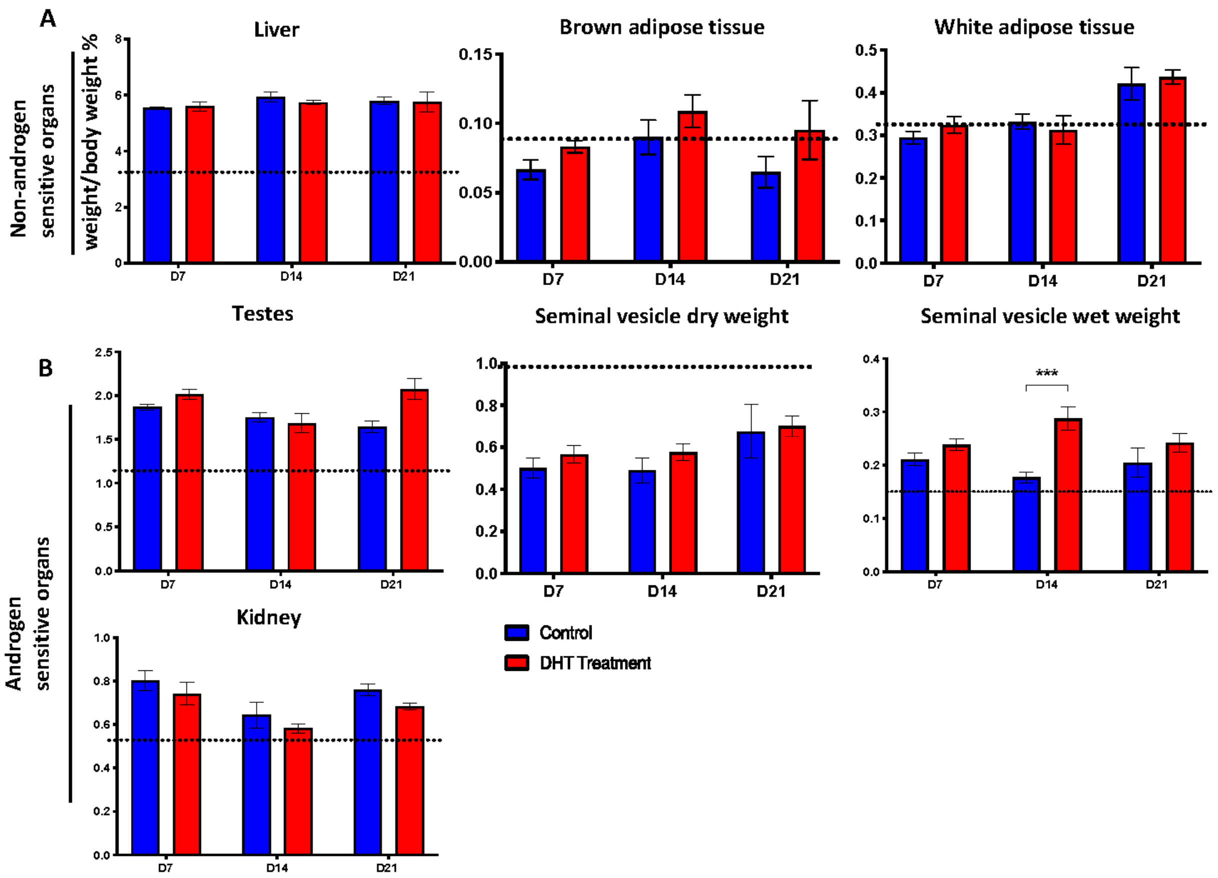

Supplemental figure 3. Androgen sensitive/non-sensitive organ weight No significant difference in weight to body weight percentage was observed for (**A**) non androgen sensitive tissues liver, brown adipose tissue and white adipose tissue at day 7, 14 and 21 post burn injury between treatment and control mice. (**B**) Androgen sensitive organs testes, kidney and seminal vesicle (dry) had similar weight to body percentage at day 7, 14 and 21 post burn injury between treatment and control mice. While, DHT treated mice had a significant increase in seminal vesicle (wet) weight to body percentage at day14 when compare to untreated mice. $N = 6$ per time point, ***$P < 0.0001$

Supplemental table 1. List of primer sequence

Gene	Accession Number	Primer Sequence
IL-6	NM_031168.2	F: TCTGCAAGAGACTTCCATCCA<br>R: AGTCTCCTCTCCGGACTTGT
TGF-β1	NM_011577.2	F: CCCGAAGCGACTACTATGC<br>R: CATAGATGGCGTTGTTGCGG
TNF-α	NM_001278601	F: TAGCCCACGTCGTAGCAAAC<br>R: GCAGCCTTGTCCCTTGAAGA<br><br>F: CCACCACGCTCTTCTGTCTA<br>R: CTTGGTGGTTTGTGAGTGTGAG
COL3α1	NM_009930.2	F: GTCCTTCAGGTGAACCCGGCA<br>R: GGAACCAGCTTCGCCCCGTT
COL1α1	NM_007742.3	F: CCAGTGGCGGTTATGACTT<br>R: GCGGATGTTCTCAATCTGC
